# Carnosic acid impedes cell growth and enhances anticancer effects of carmustine and lomustine in melanoma

**DOI:** 10.1042/BSR20180005

**Published:** 2018-07-03

**Authors:** Kun-I Lin, Chih-Chien Lin, Shyh-Ming Kuo, Jui-Chi Lai, You-Qi Wang, Huey-Ling You, Mei-Ling Hsu, Chang-Han Chen, Li-Yen Shiu

**Affiliations:** 1 Department of Cosmetic Science, Providence University, Taichung, Taiwan, R.O.C; 2 Departments of Obstetrics and Gynecology, Chang Bing Show Chwan Memorial Hospital, Lukang Zhen, Changhua County, Taiwan, R.O.C; 3 Department of Biomedical Engineering, I-Shou University, Taiwan, R.O.C; 4 Department of Biological Science and Technology, I-SHOU University, Kaohsiung, Taiwan, R.O.C; 5 Department of Laboratory Medicine, Kaohsiung Chang Gung Memorial Hospital, Kaohsiung, Taiwan, R.O.C; 6 Department of Medical Laboratory Sciences and Biotechnology, Fooyin University, Kaohsiung, Taiwan, R.O.C; 7 Guangdong Institution of Gastroenterology, Guangdong Provincial Key Laboratory of Colorectal and Pelvic Floor Diseases, The Sixth Affiliated Hospital, Sun Yat-sen University, China; 8 Department of Medical Research, E-Da Hospital, I-Shou University, Kaohsiung, Taiwan, R.O.C; 9 Cell Therapy and Research Center, Department of Medical Research, E-Da Cancer Hospital, Kaohsiung, Taiwan, R.O.C

**Keywords:** B16F10, carnosic acid, cell cycle, carmustine, lomustine

## Abstract

Carnosic acid (CA), a major polyphenolic diterpene present in *Rosmarinus officinalis*, has been reported to have multiple functions, including antitumor activity. The MTT assay, BrdU incorporation, wound healing, and colony formation were used to detect melanoma B16F10 cell growth and proliferation. Flow cytometry was used for cell cycle detection. p21 and p27 expression was detected by Western blotting. B16F10 cell xenograft model was established, and treated with CA, carmustine (BCNU), or lomustine (CCNU). The present study found that CA exhibits significant growth inhibition and cell cycle arrest in melanoma B16F10 cells. We also found that CA triggers cell cycle arrest at G_0_/G_1_ phase, and enhances p21 expression. Additionally, CA can enhance BCNU- and CCNU-mediated cytotoxicity and cell cycle arrest in B16F10 cells. Finally, we found that CA inhibits tumor growth, and reduces the values of aspartate aminotransferase (AST) and alanine aminotransferase (ALT) *in vivo*. The present study study concluded that CA may be safe and useful as a novel chemotherapeutic agent.

## Introduction

Carnosic acid (CA), which is a major polyphenolic diterpene present in rosemary (*Rosmarinus officinalis*), has been reported to have multiple biological functions, including anti-inflammation, antivirus, and anticancer [1–3]. Some studies have demonstrated that CA inhibits proliferation and migration of cancer cells [2,4–6], and also reduces vascular endothelial growth factor expression [7]. Additionally, CA induces cell cycle arrest at the G_2_/M phase via down-regulation of cyclin A expression in leukemia and colon cancer cells [8–10]. Several previous studies have reported that CA induced apoptosis in human neuroblastoma and human prostate carcinoma cells [4,11]. CA inhibits the epithelial–mesenchymal transition (EMT) and migration of B16F10 cells in a dose-dependent manner [6]. Moreover, combined treatment by CA and curcumin induces apoptosis in acute myeloid leukemia cells via a caspase-8-mediated pathway [10]. Additionally, CA enhances sensitized TRAIL-mediated apoptosis in human carcinoma caki cells [12]. Hence, combined treatment with CA and other chemotherapy drugs may be a good strategy for cancer therapy.

Carmustine (BCNU) and lomustine (CCNU) are two of the nitrosoureas belonging to classical alkylating agents. BCNU is highly soluble in alcohol and lipids, and poorly soluble in water. CCNU is soluble in absolute alcohol and is relatively insoluble in water. Just like other nitrosoureas, these two drugs can also inhibit several enzymatic processes by carbamoylation of amino acids in proteins [13,14]. The high lipid solubility of BCNU and CCNU means that they cross the blood–brain barrier quite effectively. Researchers know that BCNU and CCNU are useful to treat brain tumors as a single agent or in established combination therapy with other chemotherapeutic drugs [15–18]. However, bone marrow suppression is the most common and severe toxic effect of BCNU and CCNU [19,20]. According to a previous study, CA enhances antiproliferation activity of cisplatin on human ovarian cancer cells [21]. Cisplatin is a platinum-based chemotherapeutic drug, and frequently designated as an alkylating agent. Although cisplatin does not have an alkyl group, it nevertheless damages the DNA and interferes with DNA repair. So cisplatin is classified as an alkylating-like agent. In the present study, we tried to estimate the combined effect of CA with alkylating agents (BCNU and CCNU) on melanoma *in vitro* and *in vivo*.

Aspartate aminotransferase (AST) and alanine aminotransferase (ALT), two kinds of liver enzymes, can be found widely throughout the human body. AST is found primarily in many organs and tissues including heart, liver, skeletal muscle, and kidney. ALT is found primarily in liver and kidney [22,23]. Amongst the liver injury markers, ALT and AST are most commonly used. Liver diseases and injury are the main causes that increase ALT and AST plasma levels. Most forms of liver diseases and injury, plasma ALT levels are higher than that of AST. ALT is more specific than AST to be a liver injury marker [24]. Bilirubin is created by the breakdown of hemoglobin and passed on to the liver. Total bilirubin (TBIL) is composed of direct bilirubin and indirect bilirubin. When liver diseases or drug-induced liver injury occurs, the plasma levels of bilirubin will be higher than the normal. The liver function will be determined abnormal. High levels of bilirubin can cause jaundice, severe liver disease, and possibly cirrhosis [24]. Hence, TBIL is an important marker for liver function. In the human body, albumin (ALB) is the most abundant plasma protein, and is produced by hepatocytes. ALB is an important factor in maintaining plasma osmolality. It can be a source for other proteins synthesis. Additionally, ALB also functions as a transporter for drugs and hormones. Some diseases and injury cause plasma ALB levels to decrease, including nephrotic syndrome, burns, protein losing enteropathy, malnutrition, and liver disease [24,25]. For liver disease measurements, ALB is used as a marker to determine the progression, severity, decompensation, and prognosis of cirrhosis [26]. Therefore, current laboratory tests are important in the diagnosis and monitoring of liver injury and function. These tests focus on injury markers (AST and ALT) and functional markers (TBIL and ALB).

Melanoma is the most fatal kind of skin cancer. It is an important topic to investigate new strategies to treat cancer cells and decrease high death rates. B16F10 is a murine melanoma cell line, and is commonly used for melanoma research model. This cell line was isolated and maintained from a C57BL/6 mouse. Therefore, we use B16F10 cells and C57BL/6 mouse to estimate the treated effects of CA, BCNU, and CCNU *in vitro* and *in vivo*.

The present study investigates the regulation by CA of cell cycle distribution. CA arrests the cell cycle of B16F10 cells at the G_0_/G_1_ phase. We tested the plasma levels of AST, ALT, TBIL, and ALB after CA injection and B16F10 xenograft to evaluate liver injury and function. The combined effects of CA and BCNU or CCNU in B16F10 cells are also explored. Experimental results indicate that CA may enhance the anticancer effects of BCNU and CCNU *in vitro* and *in vivo*.

## Materials and methods

### Cell line and reagents

The melanoma cell line B16F10 used in the present study was purchased from Bioresource Collection and Research Center (BCRC; Hsinchu, Taiwan). All cells were cultured at 37°C under a 5% CO_2_ atmosphere and in Dulbecco’s modified Eagle’s medium (DMEM; Gibco®, Life Technologies™, U.S.A.) supplemented with 100 U/ml penicillin, 100 μg/ml streptomycin, and 10% heat-inactivated FBS (Gibco®, Life Technologies™, U.S.A.). CA was purchased from ChemFaces (ChemFaces, Wuhan, Hubei, China). The BCNU, CCNU, DMSO, RNase A, and propidium iodide (PI) were purchased from Sigma–Aldrich (St. Louis, MO, U.S.A.). The anti-p21, -p27, and -β-actin antibodies were purchased from GeneTex (GeneTex Inc.).

### Cytotoxicity detection

To detect the cytotoxicity of CA, BCNU, and CCNU, B16F10 cells were seeded in 96-well plates at a density of 5 × 10^3^ cells/well in DMEM supplemented with 10% FBS. Following an 18-h incubation at 37°C under a 5% CO_2_ atmosphere, the old medium was replaced with fresh DMEM/1% FBS containing CA (0, 5, 10, 15, 20, 25, 50, and 100 μM), BCNU (0, 1.57, 3.13, 6.25, 12.5, 25, 50, and 100 μM), or CCNU (0, 1.57, 3.13, 6.25, 12.5, 25, 50, and 100 μM). The MTT assay (Invitrogen, Thermo Fisher Scientific Inc.) was used to detect cytotoxicity after 24 h of incubation. The generated formazan products were solubilized by 100 μl DMSO, and the optical density was measured at 570 nm using an ELISA reader (infinite M200PRO, TECAN).

### Cell proliferation assay

The B16F10 cells were seeded on cover glasses in 24-well plates at a density of 5 × 10^4^ cells/cover glass. Following an 18-h incubation at 37°C under 5% CO_2_ atmosphere, the old medium was replaced with fresh DMEM/1% FBS containing CA (0, 1, 2, 5, 10, 15, 20, and 25 μM). Stimulation was performed for 24 h, during which BrdU was added to the medium for the last 1 h. BrdU incorporation was detected by BrdU Labeling and Detection Kit (Roche Diagnostics). The BrdU-labeled cells and nuclei were visualized by a fluorescence microscope (20×; Zeiss), and the images were analyzed by ImageJ software (National Institutes of Health, U.S.A.).

### Wound healing assay

To perform the wound healing assay, B16F10 cells were seeded in six-well culture plates at 3 × 10^5^ cells/well in DMEM/10% FBS and incubated over two nights at 37°C in 5% CO_2_ atmosphere. The cell monolayer was longitudinally scratched by a pipette tip to form a stripe. The culture medium was replaced with 1 ml fresh DMEM/1% FBS containing different concentrations of CA (0, 5, 10, and 15 μM). The cells were visualized and photographed by the inverted microscope at time 0, 12, and 24 h. The images were analyzed and quantitated by ImageJ software (National Institutes of Health, U.S.A.).

### Soft agar colony formation assay

The B16F10 cells were pre-treated with CA (0, 5, 10, and 15 μM) for 24 h. The cells were then seeded (2 × 10^3^ cells/well) into a six-well plate containing DMEM plus 10% FBS and CA (0, 5, 10, and 15 μM) in 0.4% agar above a layer of 0.6% agar. The cells were incubated at 37°C under a 5% CO_2_ atmosphere for 14 days, and the colonies were photographed and counted under an optical microscope (CKX41, OLYMPUS).

### Cell cycle detection

The cell cycle was detected by treating B16F10 cells with CA (0, 2, 5, 10, and 15 μM) for 24 h. All cells were fixed in 70% ethanol/phosphate-buffered saline (PBS) at 4°C for 18 h, pelleted, and then resuspended in PBS containing 200 μg/ml RNase A and 0.01 mg/ml PI. The cell cycle was determined by flow cytometry (Cytomic FC 500, Beckman Coulter).

### Western blotting

The B16F10 cells were seeded in 12-well plates at a density of 2 × 10^5^ cells/well. The cells were treated with drugs for 24 h after 16 h of incubation. The cells were subsequently lysed in RIPA buffer (G-Biosciences, MO, U.S.A.) containing protease inhibitors (Roche Applied Science, Mannheim, Germany). The total extracted protein content (25 μg) was separated by SDS/PAGE, and transferred on to PVDF membranes. The PVDF membranes were incubated with primary antibodies at a dilution of 1:500 or 1:1000 to detect p21 (catalog number: GTX27960, GeneTex Inc., U.S.A.), p27 (catalog number: GTX55090, GeneTex Inc., U.S.A.), and β-actin (catalog number: GTX109639, GeneTex Inc., U.S.A.). The protein band density was quantitated by ImageJ software (National Institutes of Health, U.S.A.). The fold change in protein expression was expressed as a ratio calculated by dividing the p21 or p27 protein band density by the β-actin band density and then normalized to the control group.

### Ethics approval and consent to participate

The present study was performed with standard operation protocols. The animal experiments were approved by the Institutional Animal Care and Use Committee (IACUC) of E-Da Hospital (approval number: IACUC-103033).

### Mouse xenograft model

Male C57BL/6 mice at 6–8 weeks of age were purchased from BioLASCO Taiwan Co., Ltd. The B16F10 cells (5 × 10^5^ in 0.1 ml of PBS) were injected subcutaneously into the backs of the mice. Seven days after B16F10 cell injection, all mice were separated randomly into six groups: control (0.1 ml PBS) group, CA (50 mg/kg) treated group, BCNU (35 mg/kg) treated group, CCNU (50 mg/kg) treated group, CA + BCNU treated group, and CA + CCNU treated group. PBS or drugs were injected peritoneally every 2 days (*n*=5 for each group). The largest diameter and smallest diameter of the tumor were measured and the tumor volume (mm^3^) was calculated as largest diameter (mm) × smallest diameter^2^ (mm^2^)/2. Peripheral blood of mice was also accumulated. The amounts of AST, ALT, TBIL, and ALB in peripheral blood was measured by UniCel™ DxI 800 Immunoassay System (Beckman Coulter).

### Statistical analysis

The data are presented as S.D. of three determinations (means ± S.D., *n*=3). The statistical significance of the differences between pairwise comparisons was determined using the unpaired Student’s *t* test. *P*<0.01 was considered statistically significant.

## Results

### CA inhibited cell growth in melanoma B16F10 cells

**Figure 1:**
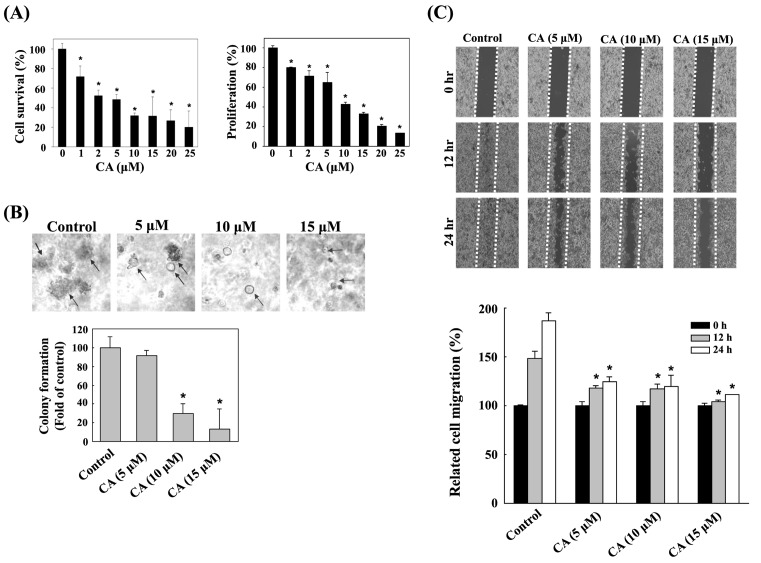
CA inhibited cell survival, proliferation, colony formation, and wound healing activity of B16F10 cells (**A**) B16F10 cells were treated with serial concentrations of CA for 24 h. The cell survival and proliferation were detected by MTT assay and BrdU incorporation. Data are means ± S.D. from three independent tests. *P<0.01 compared with the 0 μM group. (**B**) B16F10 cells were pre-treated with CA for 24 h. The colony formation assay was performed to detect cell growth. Data are means ± S.D. from five independent experiments. *P<0.01 compared with the control group. (**C**) B16F10 cells were seeded into a six-well plate. The cells were treated with CA after two-night incubation. The cells were photographed at time 0, 12, and 24 h. The images were quantitated by ImageJ software. CA inhibited wound healing activity of B16F10 cells. Data are means ± S.D. from three independent tests. *P<0.01 compared with the control group.

To explore the cytotoxic effect of CA, B16F10 cells were treated with CA (0, 1, 2, 5, 10, 15, 20, and 25 μM) for 24 h. Cell viability was measured by MTT assay. Cell proliferation was determined by BrdU incorporation. Experimental results indicate that CA significantly inhibited cell viability and proliferation of B16F10 cells (Figure 1A). The IC_50_ values of CA were calculated from the results of MTT and BrdU assays were approximately (7.08 ± 0.14) μM and (8.64 ± 1.02) μM, respectively. Additionally, soft agar colony formation assay and wound healing assay were adopted to detect cell growth of B16F10 cells. As indicated in Figure 1B,C, CA inhibited colony formation and wound healing of B16F10 cells significantly after incubation with CA (5, 10, and 15 μM). Moreover, flow cytometry was used to detect cell cycle of B16F10 cells following CA 24-h treatment. The percentage of G_0_/G_1_ phase increased significantly (Table 1). These data indicate that CA may inhibit cell growth and induce G_0_/G_1_ phase arrest in B16F10 cells.

**Table 1:** CA arrested B16F10 cells at G_0_/G_1_ phase after 24-h treatment

	CA				
Cell cycle	0 μM	2 μM	5 μM	10 μM	15 μM
**Sub-G_1_ (%)**	0.4 ± 0.1	0.8 ± 0.2	0.5 ± 0.4	0.5 ± 0.1	0.7 ± 0.2
**G_0_/G_1_ (%)**	65.7 ± 3.1	85.5 ± 2.7*	86.3 ± 5.1*	92.7 ± 2.9*	85.1 ± 6.1*
**S (%)**	21.4 ± 1.7	7.7 ± 0.9	7.1 ± 3.1	3.3 ± 0.1	5.0 ± 0.8
**G_2_/M (%)**	12.9 ± 0.7	6.1 ± 1.6	6.1 ± 0.4	3.8 ± 0.4	9.5 ± 1.5

*n*=3.

* *P*<0.01 and comapred with the 0 μM group.

### CA enhanced the cytotoxic effects of BCNU and CCNU in B16F10 cells

**Figure 2:**
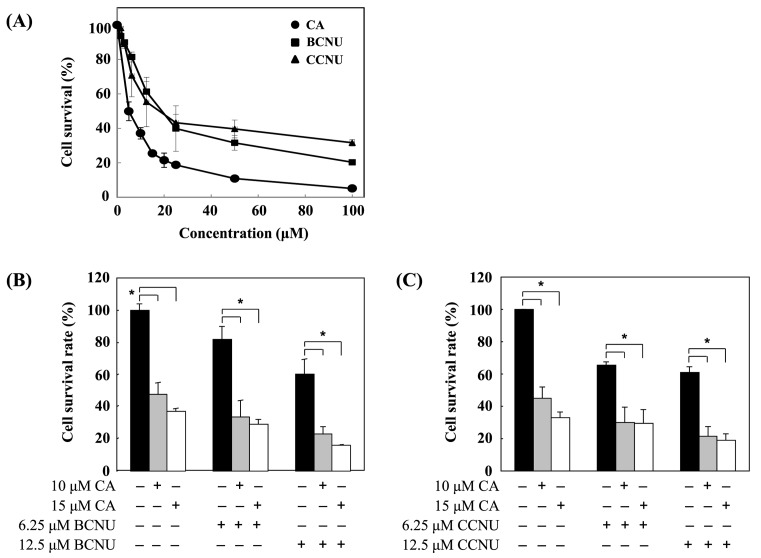
CA enhanced BCNU- and CCNU-induced cytotoxicity on B16F10 cells (**A**) B16F10 cells were seeded in 96-well plates at a density of 5 × 10^3^ cells/well. The cells were treated with CA, BCUN, and CCNU for 24 h after overnight incubation. The cytotoxicity was detected by MTT assay. (**B**,**C**) B16F10 cells were treated with CA, BCNU, and CCNU alone or in combination. The cytotoxicity was also detected by MTT assay. Data are means ± S.D. from three independent tests. *P<0.01.

To compare the cytotoxicity of CA, BCNU, and CCNU, B16F10 cells were treated with CA, BCNU, and CCNU for 24 h. The MTT assay was used to detect cell survival rate. CA had a better cytotoxic effect than BCNU or CCNU (Figure 2A). The combined effects of CA and BCNU or CCNU were determined. B16F10 cells were treated with CA alone, BCNU alone, CCNU alone, CA plus BCNU or CA plus CCNU. Cell survival rate and cell cycle distribution were detected by MTT assay and flow cytometry, respectively. As shown in Figure 2B,C, CA enhanced the cytotoxic effects of BCNU and CCNU. CA increased BCNU- or CCNU-induced cytotoxicity in B16F10 cells. Additionally, cell cycle distribution was detected by flow cytometry after PI staining. CA arrested cell cycle at G_0_/G_1_ phase, and BCNU and CCNU arrested cell cycle at G_2_/M phase. Additionally, 15 μM of CA enhanced BCNU- or CCNU-induced G_2_/M phase arrest (Tables 2 and 3).

**Table 2:** CA enhanced BCNU-induced G_2_/M phase arrest

	Sub-G_1_	G_0_/G_1_	S	G_2_/M
**Control**	0.7 ± 0.15	69.4 ± 1.47	16.2 ± 0.84	10 ± 0.14
**CA (15 μM)**	0.4 ± 0.24	78.7 ± 1.89	8.6 ± 1.35	10.5 ± 0.71
**BCNU (12.5 μM)**	0.7 ± 0.12	60.4 ± 3.17	17.4 ± 1.6	21 ± 0.23
**CA (15 μM) + BCNU (12.5 μM)**	0.3 ± 0.07	46.4 ± 1.83	15.7 ± 2.11	37.6 ± 1.52*

*n*=3. *P<0.01 compared with the control, CA or BCNU group.

**Table 3:** CA enhanced CCNU-induced G_2_/M phase arrest

	Sub-G_1_	G_0_/G_1_	S	G_2_/M
**Control**	0.7 ± 0.15	69.4 ± 1.47	16.2 ± 0.84	10 ± 0.14
**CA (15 μM)**	0.4 ± 0.24	78.7 ± 1.89	8.6 ± 1.35	10.5 ± 0.71
**CCNU (12.5 μM)**	1 ± 0.16	55 ± 2.36	18 ± 3.45	25.7 ± 2.01
**CA (15 μM) + CCNU (12.5 μM)**	0.1 ± 0.05	56.6 ± 2.73	12.5 ± 1.63	30.5 ± 1.41^*,†^

*n*=3. *P<0.01 compared with the control or CA group. ^†^*P*< 0.05 compared with the CCNU group.

### CA regulated p21 and p27 protein expression

**Figure 3:**
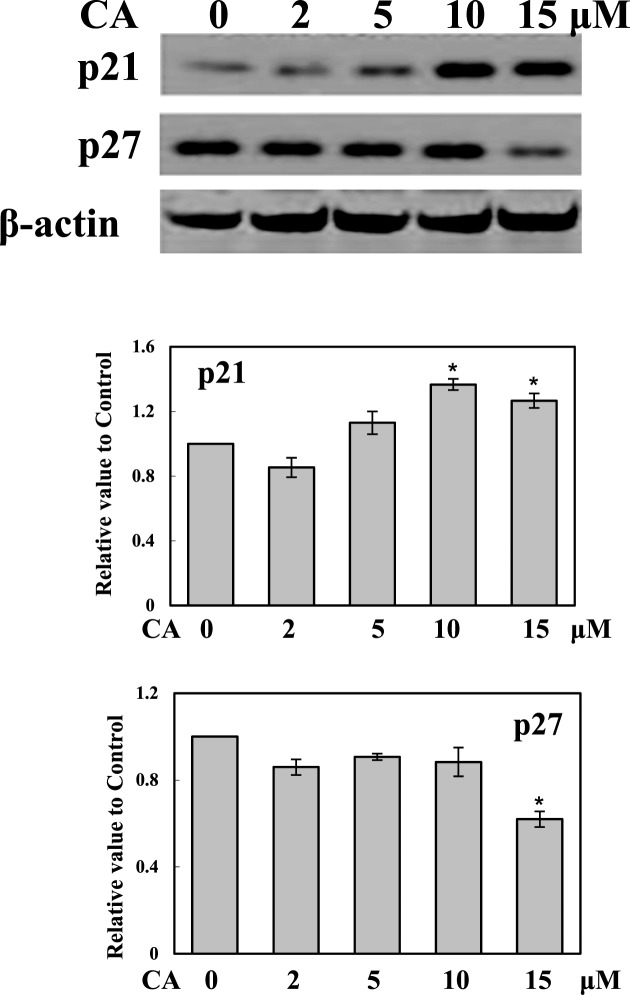
CA increased p21 expression and decreased p27 expression B16F10 cells were treated with or without CA (2, 5, 10, and 15 μM) for 24 h. The total protein was extracted, and 25 μg proteins were separated with SDS/PAGE. Western blot analysis was performed to detect p21 and p27 expression. The protein band density was quantitated by ImageJ software. *P<0.01 compared with the 0 μM group.

The expression levels of p21 and p27 were further detected. Researchers have previously reported that p21 and p27 play critical roles in cell cycle regulation. Up-regulation of p21 and p27 in many cancer cells can arrest cell cycle at G_0_/G_1_ and G_2_/M phase. To investigate the regulating effects of CA on p21 and p27, total protein was extracted from B16F10 cells with RIPA buffer after CA (2, 5, 10, and 15 μM) 24-h treatment. As shown in Figure 3, CA up-regulated p21 expression and down-regulated p27 expression of B16F10 cells within 24 h.

### CA inhibited B16F10 tumor growth and enhanced antitumor effects of BCNU and CCNU

**Figure 4:**
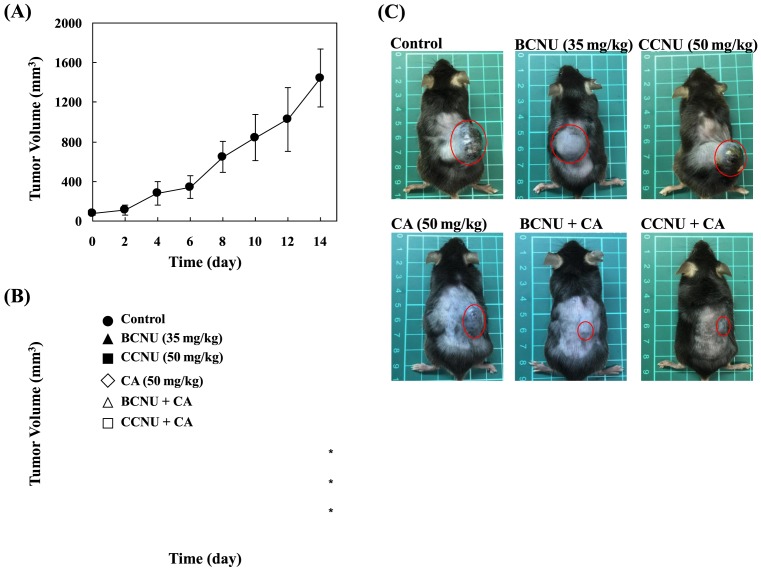
CA inhibited melanoma tumor growth and enhanced the antitumor effects of BCNU and CCNU *in vivo* (**A**) B16F10 cells were injected subcutaneously on the back of C57BL/6 mice (*n*=5). After 7 days, the tumor size was measured every 2 days. (**B**) After B16F10 tumor xenograft, total mice were separated into six groups: control, BCNU treated (35 mg/kg), CCNU treated (50 mg/kg), CA treated (50 mg/kg), BCNU + CA treated, and CCNU + CA treated. The mice were treated every 2 days. The tumor volume was measured and calculated. *n*=5; *P<0.01 compared with the control group. (**C**) After a 14-day treatment (treated seven times), the mice were killed and photographed. The red circle indicates the tumor location site.

The B16F10 cells were xenografted to the back of C57BL/6 mice. After 7 days, the tumor size was measured every 2 days (Figure 4A). After 14 days, the tumors grew markedly, and the values of AST and ALT were enhanced significantly. However, the tumor growth did not significantly affect TBIL or ALB values (Table 4).

**Table 4:** The liver function tests after B16F10 xenograft

	AST (IU/l)	ALT (IU/l)	TBIL (mg/dl)	ALB (g/l)
**Day 0**	112.4 ± 15.32	30.8 ± 9.31	0.54 ± 0.29	2.66 ± 0.25
**Day 14**	1153 ± 178.87*	140.2 ± 0.32*	0.44 ± 0.21	3.02 ± 0.31

*n*=5. *, *P*<0.01 compared with the day 0 group.

**Figure 5:**
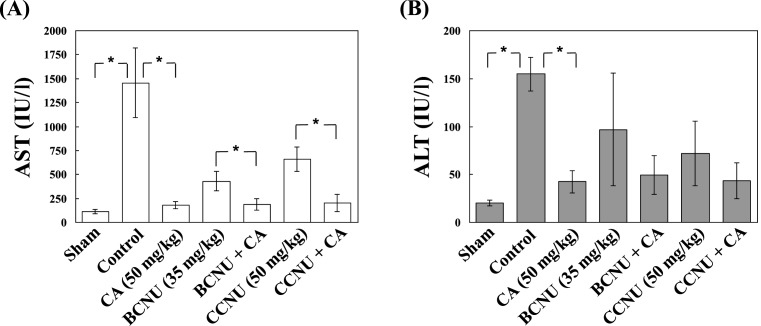
CA decreased the levels of AST and ALT of C57BL/6 mice The whole blood of mice was collected after 14-day treatment with CA or other drugs. The values of AST (**A**) and ALT (**B**) were measured by UniCelTM DxI 800 Immunoassay System. The mice in the sham group were healthy, and were not experimented. The mice in the control group received vehicle (PBS) injection peritoneally after tumor xenograft. The control group had much higher AST and ALT values than any other groups. CA decreased the AST and ALT values significantly, on its own and in BCNU + CA and CCNU + CA treated groups. *n*=5; *P<0.01.

The normal mice were injected with CA (50 mg/kg) peritoneally every day for 7 days before antitumor effect determination. The CA did not significantly increase AST, ALT, TBIL, or ALB values (Table 5). These data suggest that CA does not injure the liver function of mice. To determine antitumor effects of CA, the mice were divided into six groups randomly, as described in the ‘Materials and methods’ section. Experimental results indicate that CA had a better antitumor effect than BCNU or CCNU. Additionally, CA significantly enhanced antitumor effects of BCNU and CCNU (Figure 4B,C). Moreover, the control group had much higher AST levels (1456.4 ± 360.6 IU/l) than other groups. The AST levels of the sham, CA, BCNU, CCNU, CA + BCUN, and CA + CCNU groups were (113.16 ± 24) IU/l, (180.1 ± 34.4) IU/l, (430.2 ± 102.5) IU/l, (657.8 ± 128.1) IU/l, (187.3 ± 60.3) IU/l, and (201.7 ± 88.8) IU/l, respectively. The AST values were significantly reduced in CA, CA + BCUN, and CA + CCNU treated groups. The ALT levels of the sham, control, CA, BCNU, CCNU, CA + BCUN, and CA + CCNU group were (20.3 ± 3) IU/l, (154.8 ± 17.8) IU/l, (42.45 ± 11.7) IU/l, (97 ± 58.7) IU/l, (72 ± 34) IU/l, (49.3 ± 20.2) IU/l, and (43.5 ± 18.5) IU/l, respectively (Figure 5). These data suggest that CA may be safe and useful for melanoma treatment in the future. In addition, CA may be suitable for BCNU or CCNU co-treatment in clinics.

**Table 5:** The liver function tests after CA (50 mg/kg) injection

	AST (IU/l)	ALT (IU/l)	TBIL (mg/dl)	ALB (g/dl)
**Day 0**	116.6 ± 17.17	30.2 ± 10.52	0.6 ± 0.16	2.54 ± 0.21
**Day 7**	114.8 ± 10.69	29.4 ± 7.3	0.5 ± 0.19	2.8 ± 0.22

*n*=5.

## Discussion

CA is one of the main pharmacological compounds extracted from rosemary (*R. officinalis*). CA displays some biological functions, including antioxidant, anti-inflammatory, and anticancer [1–3]. Some previous studies have shown that CA inhibits cancer cell proliferation and migration [2,4–6]. CA arrests cell cycle at the G_2_ phase of human glioma cells [27]. p21 is a cyclin-dependent kinase inhibitor that triggers apoptosis, cell cycle arrest, and proliferative inhibition in response to many stimulations. p21 is regulated by p53, and plays important roles in inhibiting cyclin-dependent kinases such as CDK2, CDK3, CDK4, and CDK6, leading to G_0_/G_1_ cell cycle arrest [28,29]. However, p21 can also block the cell cycle at the G_2_/M phase through interaction with PCNA and CDK1 [30]. p27, which is a key tumor suppressor similar to p21, is another important cell cycle progression regulator. Down-regulation of both p21 and p27 expression in cancer cells result in more aggressive tumors [31]. p27 arrests the cell cycle at the G_0_/G_1_ phase by inhibiting CDK/cyclin complexes [32,33]. Both reduction and loss of p27 expression lead to cancer cell proliferation and increased invasiveness. The present study found that CA inhibited B16F10 cell survival, proliferation, colony formation and, wound healing activity (Figure 1). We also found that CA arrests B16F10 cells at the G_0_/G_1_ phase, and that the most effective dose of CA was 10 μM (Table 1). Additionally, CA increased p21 expression significantly in a dose-dependent manner. CA but decreased p27 expression while at treated doses over 10 μM (Figure 2), thus possibly explaining why CA had the strongest effect on the cell cycle at a concentration of 10 μM.

Melanoma, one of the most aggressive malignancies, exhibits low response rates of standard therapy, and has a constancy of poor prognosis. Combined treatment strategies are commonly used in clinics. For example, BCNU is administered alone or in combination with other drugs, including darcabazine and cisplatin. Another combined treatment in common use for melanoma is the CVD regimen, comprising BCNU, vinblastine, and dacarbazine [34]. CCNU is also used in the other combined strategy. For example, CCNU combined with bleomycin, vincristine, and dacarbazine (as a BOLD regimen) can be used to treat patients with metastatic uveal melanoma [35]. Taken together, BCNU and CCNU are suitable for combined therapy in clinics. The present study used B16F10 cells to set up a melanoma tumor xenograft model, and treated mice with CA, BCNU, CCNU, CA + BCNU, or CA + CCNU. The combined treatment of CA with BCNU or CCNU (CA + BCNU and CA + CCNU groups) was more effective than any single treatment by these drugs (Figure 3). These two combined strategies may be useful in future clinical applications.

Normally, AST can be detected in red blood cells, muscle, liver, heart and, other organs. Damaged tissues or organs cause AST to be released into the bloodstream. High plasma levels of AST can be directly detected. Therefore, AST can be used as an indicator of tissue damage. The other important liver enzyme is ALT, which is more specific for the liver than AST. The blood tests for AST and ALT are often performed at the same time. These blood tests are considered to be two of the most important tests to determine liver damage. The ratio of AST and ALT is used to distinguish liver injury from damage to the muscle, heart, or other organs. Most of liver disease cases have a ratio of AST/ALT less than 1. In contrast, heart or muscle injury generally has higher levels of AST than ALT, with a ratio of AST/ALT much greater than 1. In the present study, the average AST value in the control group was greater than 1000 IU/l, which was higher than in other treated groups (Table 4 and Figure 5A). Additionally, the tumor volume was found to be positively correlated with AST value. Melanoma tumor also caused the ALT value to increase. However, the ratio of AST/ALT of control group was much higher than 1 (Table 4 and Figure 5). These results indicate that the melanoma tumor may corrupt the muscle layer of mice, but does not damage liver harmfully. CA or combined treatments significantly decreased the AST value. These data indicate that CA may inhibit melanoma tumor growth and erosion, and enhance the antitumor effects of BCNU and CCNU. A biosafety test of CA was also performed in the present study. A single CA injection (50 mg/kg, injected peritoneally every day for 7 days) did not increase AST, ALT, TBIL, or ALB values significantly (Table 5). Even raising the injected CA dose to 100 mg/kg still did not cause obvious damage to the mice (data not shown), indicating that CA may be safe to use in both animal and human bodies.

In conclusion, CA inhibits melanoma cell growth and arrests cell cycle significantly. Additionally, CA inhibits melanoma tumor growth, and improves the anticancer effects of BCNU and CCNU *in vitro* and *in vivo*. Finally, the present study indicates that CA may be safe to use in future clinical trials.

## Abbreviations

ALB: albumin
ALT: alanine aminotransferase
AST: aspartate aminotransferase
BCNU: carmustine
CA: carnosic acid
CCNU: lomustine
DMEM: Dulbecco’s modified Eagle’s medium
PI: propidium iodide
TBIL: total bilirubin
